# COVID-19 in heart transplant recipients

**DOI:** 10.34172/jcvtr.2022.31583

**Published:** 2022-12-17

**Authors:** Sepideh Taghavi, Hoda Raffiei Jelodar, Ali Rafati, Nasim Naderi, Marzieh Mirtajaddini, Ahmad Amin, Leili Valizadeh, Razieh Omidvar, Monireh Kamali, Soroush Naseh

**Affiliations:** ^1^Heart Valve Disease Research Center, Rajaie Cardiovascular Medical and Research Center, Iran University of Medical Sciences, Tehran, Iran; ^2^Rajaie Cardiovascular Medical and Research Center, Iran University of Medical Sciences, Tehran, Iran; ^3^University of Auckland, Auckland, New Zealand

**Keywords:** SARS-CoV-2, COVID-19, Heart Transplantation, Immunosuppressive Medication

## Abstract

***Introduction:*** After solid organ transplantation, patients require lifelong immunosuppressive medication, increasing susceptibility to COVID-19. We evaluated the clinical outcomes of heart transplant recipients in patients with COVID-19.

***Methods:*** We enrolled twenty-two COVID-19 cases of adult heart transplantation from February 2020 to September 2021.

***Results:*** The most common symptoms in patients were fever and myalgia. The death occurred in 3 (13.6 %).

***Conclusion:*** Although heart transplantation mortality may increase in the acute rejection phase concomitant with COVID-19, immunosuppressive dose reduction may not be necessary for all heart transplant patients with COVID-19.

## Introduction

 The severe acute respiratory syndrome-coronavirus-2 (SARS-CoV-2) pandemic has posed unprecedented health challenges. Patients receiving solid organ transplantation require lifelong immunosuppression, thus more susceptible to COVID-19.^1^ There are few data on the prevalence and clinical features of COVID-19 in heart transplant patients. Heart transplant recipients have comorbidities such as diabetes mellitus (DM), hypertension (HTN), chronic kidney disease (CKD), obesity, coronary heart disease, coronary allograft vasculopathy (CAV), and chronic lung diseases, making them prone to severe diseases.^2^ While managing immunosuppressive medication in individuals with severe COVID-19 is uncertain,^3^ the relationship between COVID-19 and heart transplant rejection needs attention. Therefore, we evaluated the clinical outcomes of heart transplantation in patients with confirmed COVID-19.

## Materials and Methods

###  Study design

 We conducted observational research at Rajaie Heart Center (RHC), Iran. From February 2020 to September 2021, this research included all adult heart transplant patients with positive nasopharyngeal reverse transcriptase-polymerase chain reaction (RT-PCR) testing for SARS-CoV-2.

 We gathered data from medical records on hospitalized patients and outpatients. Patients’ clinical and laboratory data, immunosuppression, and early antiviral treatments were documented.

 Patients were classified as having a mild infection (requiring just outpatient treatment), a moderate infection (requiring admission to the general inpatient ward), or a severe disease (mechanical ventilation, intensive care unit (ICU) admission, or death).

###  Statistical analysis

 Continuous variables were expressed as mean ± standard deviation. Categorical data were provided as frequency and were compared using the Chi-square test. Significance was defined as a two-sided p-value of < 0.05. SPSS software version 24 was used for analysis.

## Results

 The detailed characteristics of the patients are shown in Table 1. Table 2 summarizes on-admission characteristics, risk factors, and laboratory data. Although SARS-CoV-2 PCR was negative in five individuals, a chest computed tomography (CT) scan suggested COVID-19.

**Table 1:** Characteristics of the patients.

**Patients**	**Age, years after transplant**	**Admission**	**Risk factors**	**Symptoms**	**Immunosuppression therapy **	**Rejection in COVID-19 infection**	**COVID-19 treatment**	**Adverse effects **	**Immunosuppression dosage change**	**Intubation, Death**
1	34, 4	Yes	DM, CKD, Obesity	dyspnea, fever, nausea and vomiting	prednisolone, mycophenolate mofetil, sirolimus, cyclosporine	Yes	prednisolone, IVIG, ATG	No	increased prednisolone (methylprednisolone pulse) and mycophenolate mofetil^*^	No, No
2	18, 2	Yes	Obesity, DM, HTN	Myalgia and bone pain, fever	prednisolone, mycophenolate mofetil, tacrolimus	No	remdesivir, dexamethasone	No	increased dexamethasone	No, No
3	42, 5	No	Obesity	fever, myalgia, and bone pain	prednisolone, mycophenolate mofetil, tacrolimus	No	No	No	No	No, No
4	32, 2	Yes	HTN	dyspnea, myalgia and bone pain, fever	prednisolone, mycophenolate mofetil, tacrolimus	No	remdesivir, dexamethasone	No	No	No, No
5	31, 4	Yes	No	fever, myalgia and bone pain	prednisolone, mycophenolate mofetil, tacrolimus, sirolimus	No	remdesivir, dexamethasone	No	decreased sirolimus	No, No
6	51, 2	Yes	HTN, DM, CKD	cough, fever, myalgia and bone pain	prednisolone, mycophenolate mofetil, cyclosporine	No	remdesivir, dexamethasone	Yes (leukopenia)	decreased mycophenolate mofetil, GCSF added	No, No
7	16, 2	Yes	DM, Obesity, HTN	cough, dyspnea, fever	mycophenolate mofetil, tacrolimus	Yes	methylprednisolone	No	increased tacrolimus and mycophenolate mofetil^*^	Yes, Yes^†^
8	33, 2	Yes	DM	abdominal pain, nausea and vomiting	prednisolone, mycophenolate mofetil, tacrolimus	Yes	methylprednisolone	No	increased tacrolimus	No, Yes^†^
9	45, 3	Yes^‡^	Obesity, HTN	atypical chest pain, fever, myalgia and bone pain	mycophenolate mofetil, tacrolimus	No	prednisolone	No	increased tacrolimus	No, No
10	43, 4	Yes	Anemia, DM, CKD	fever, myalgia and bone pain, cough	mycophenolate mofetil, cyclosporine	No	remdesivir, dexamethasone	Yes (leukopenia)	decreased mycophenolate mofetil, GCSF added	No, No
11	30, 6	Yes	DM, HTN, Obesity, CKD	fever, chills, myalgia and bone pain, weakness, dyspnea, nausea and vomiting	prednisolone, mycophenolate mofetil, cyclosporine, sirolimus	No	remdesivir, dexamethasone	Yes (creatinine rise, leukopenia)	Sirolimus discontinued, decreased mycophenolate mofetil, decreased cyclosporine	No, No
12	23, 5	Yes	CAV	weakness, abdominal pain, nausea and vomiting	prednisolone, mycophenolate mofetil, sirolimus	No	remdesivir, prednisolone	No	increased prednisolone	No, No
13	42, 3	Yes	Obesity	fever, myalgia and bone pain	prednisolone, mycophenolate mofetil, tacrolimus	No	remdesivir, dexamethasone	Yes (leukopenia)	decreased mycophenolate mofetil, GCSF added	No, No
14	39, 3	Yes^‡^	Obesity, DM	cough, fever, myalgia and bone pain, dyspnea, nausea and vomiting, abdominal pain	prednisolone, mycophenolate mofetil, tacrolimus, sirolimus	No	remdesivir, dexamethasone	Yes (leukopenia)	decreased sirolimus and mycophenolate mofetil	No, No
15	33, 2 weeks	Yes	DM	No symptom	prednisolone, mycophenolate mofetil, tacrolimus	No	No	No	No	No, No
16	39, 2 months	Yes	CKD	dyspnea	mycophenolate mofetil, tacrolimus	Yes	IVIG, hydrocortisone, hydroxyl chloroquine	Yes (leukopenia, liver failure)	mycophenolate mofetil and tacrolimus discontinued	Yes, Yes
17	37, 2	Yes	Obesity	dyspnea, fever, myalgia and bone pain	prednisolone, mycophenolate mofetil, tacrolimus	No	remdesivir, dexamethasone	Yes (leukopenia)	decreased mycophenolate mofetil, GCSF added	No, No
18	39, 4	No	DM	myalgia and bone pain, abdominal pain	prednisolone, mycophenolate mofetil, tacrolimus	No	remdesivir	No	No	No, No
19	35, 3	No	No	cough	prednisolone, mycophenolate mofetil, cyclosporine	No	No	No	decreased mycophenolate mofetil	No, No
20	30, 3	Yes	No	fever, myalgia and bone pain, cough	prednisolone, mycophenolate mofetil, tacrolimus	No	dexamethasone	No	No	No, No
21	33, 3	Yes	Obesity	fever, myalgia and bone pain, cough	mycophenolate mofetil, tacrolimus	No	remdesivir, dexamethasone	No	No	No, No
22	34, 2	Yes	HTN	fever, myalgia and bone pain, abdominal pain	prednisolone, mycophenolate mofetil, tacrolimus	No	remdesivir, dexamethasone	No	decreased tacrolimus	No, No

Abbreviations: ATG, anti-thymocyte globulin; CAV, coronary allograft vasculopathy; CKD, chronic kidney disease; DM, diabetes mellitus; HTN, hypertension; GCSF, granulocyte colony-stimulating factor; IVIG, intravenous immunoglobulin. ^*^ increased mycophenolate mofetil dosage due to acute rejection. ^†^acute rejection. ^‡^experienced two episodes of COVID-19.

**Table 2:** On-admission characteristics, risk factors, and laboratory data.

**Variables**	**Mean±SD**
Age (years)	34.95 ± 8.97
BMI (kg/m^2^)	26.23 ± 5.2
Years after transplant	3.09 ± 1.57
Hypertension^*^	7 (31.8%)
Chronic kidney disease^*^	5 (22.7%)
Obesity (BMI > 25)^*^	10 (45.4%)
Diabetes Mellitus ^*^	10 (45.4%)
Mycophenolate mofetil use ^*^	22 (100%)
Tacrolimus use ^*^	16 (72.7%)
Cyclosporine use ^*^	5 (22.7%)
Sirolimus use ^*^	5 (22.7%)
Ejection fraction (%)	42.5 ± 1.2
FBS (mg/dL)	125.55 ± 47.2
Albumin (g/L)	36.9 ± 11.46
D-dimer (μg/mL)	1.10 ± 1.5
Troponin (ng/dL)	0.67 ± 1.35
Pro-BNP (pg/mL)	342.5 ± 608.62
CRP (mg/dL)	32.22 ± 44.37
WBC (cell/mm^3^)	7449.47 ± 6211.51
Hemoglobin (g/dL)	12.52 ± 2.73
Platelet (10^3^/mm^3^)	175.94 ± 56.83
Blood urea nitrogen (mg/dL)	17.84 ± 8.54
Creatinine (mg/dL)	1.23 ± 0.49
AST (IU/L)	33.52 ± 31.72
ALT (IU/L)	41.68 ± 57.78
ALP (IU/L)	147.76 ± 75.04
LDH (IU/L)	571.1 ± 309.81
Bilirubin (mg/dL)	1.72 ± 1.59

Abreviations: ALP, alkaline phosphatase; ALT, alanine transaminase; AST, aspartate transaminase; BMI, body mass index; BNP, brain natriuretic peptide; CRP, c-reactive protein; FBS, fasting blood sugar; IU, international unit; LDH, lactate dehydrogenase; SD, standard deviation; WBC, white blood cell. -All values are reported as mean ± SD unless otherwise stated. * Number (%).

 Fever (16 (72.7%)) was the most common symptom in our patients. A summary of the patents’ outcomes is demonstrated in Table 3. Three patients (13.6%) died in this research (Table 3), one with severe gastrointestinal complications (patient 8) and suspicion of acute rejection. He died of sudden cardiac death one day after discharge. Another patient suspected of acute rejection and experiencing cough and dyspnea was treated with methylprednisolone. Three days after discharge, he was readmitted with COVID-19 to the ICU and ultimately expired (patient 7). The last patient, with early COVID-19 after the transplant, had a severe clinical course with sepsis and multiple end-organ failures, which led to death (patient 16) (Table 1).

**Table 3:** Patients’ outcomes and adverse events.

**Variables**	**Number of patients, n (%)**
Hospitalized patients	19 (86.36%)
Fever	16 (72.7%)
Myalgia and bone pain	15 (68.2%)
Cough	7 (31.8%)
Dyspnea	7 (31.8%)
Abdominal pain	5 (22.7%)
Positive troponin	5 (22.7%)
Positive Pro-BNP (Pro-BNP > 125ng/dl)	6 (27.3%)
Moderate to severe right ventricular dysfunction	6 (27.3%)
Pericardial effusion	2 (9%)
Change in immunosuppression therapy	16 (72.7%)
Steroid treatment for COVID-19	18 (81.8%)
Remdesivir use	13 (59%)
GCSF due to leukopenia	4 (18.2%)
Rejection during COVID-19 infection	4 (18.1%)
Intubation and ICU admission	2 (9%)
Death	3 (13.6%)

Abreviations: GCSF, granulocyte colony-stimulating factor; ICU, intensive care unit.

 Due to severe leukopenia, immunosuppressive discontinuation and mycophenolate mofetil dosage reduction was required in 2 (9%) and 7 (31.8%) patients, respectively (Table 1).

 The COVID-19 risk factors were not significantly different between survivors and non-survivors (Table 4).

**Table 4:** Comparison of the risk factors in COVID-19 non-survivors and survivors.

**Risk factors**	**COVID-19 non-survivors (n=3), n (%)**	**COVID-19 survivors (n=19), n (%)**	***P*** ** Value**
Diabetes mellitus	2 (66.7%)	9 (47.36%)	0.534
Hypertension	1 (33.3%)	6 (31.57%)	0.951
Obesity	1 (33.3%)	9 (47.36%)	0.650
Chronic kidney disease	1 (33.3%)	4 (21%)	0.222

The analyses were performed using the Chi-square test. *P* value < 0.05

## Discussion

 COVID-19 is more common in solid organ transplant recipients compared to the general population. This increased prevalence is probably due to increased susceptibility to infections due to their chronic use of immunosuppressants.^4^

 Our study’s mortality rate (13.6%) was lower compared to previous studies (29.7%, 28.75%, and 22.7% in the studies by Bottio et al, Rivinius et al and Singhvi et al respectively).^4-6^ This could be due to the higher prevalence of risk factors or older patients in the mentioned studies.

 Pereira et al evaluated 90 individuals undergoing solid organ transplantation with COVID-19, 9 of whom were heart transplant recipients. Overall, 76% of patients were hospitalized, and 18% died. The mortality rate was similar, but ICU admission was lower than ours, possibly because their study was conducted in the early days of COVID-19, in which the appropriate treatments were not widely known.^7^

 In the present study, only 2 patients (9%) required immunosuppressive discontinuation due to sepsis and severe leukopenia, while 7 patients (31.8%) required mycophenolate mofetil dosage reduction due to leukopenia. Even though we did not reduce the dosage in other patients, we witnessed a reduced death rate. As a result, maintaining antimetabolite dosage in individuals without leukopenia seems reasonable. Discontinuation of these medicines solely due to COVID-19, without adverse effects or complications, is not recommended.

## Conclusion

 As RHC is a large tertiary heart transplant center, we had a high rate of COVID-19 in our transplant patients. Our patients were younger than in other studies, and the other risk factors were less prevalent. This may explain why our patients had lower rates of mortality and ICU admission

## Acknowledgements

 We would like to thank the participants of this study.

## Author Contributions

**Conceptualization: **Sepideh Taghavi.

**Methodology: **Sepideh Taghavi, Nasim Naderi, Hoda Raffiei Jelodar.

**Validation:** Marzieh Mirtajaddini, Ahmad Amin.

**Formal Analysis: **Hoda Raffiei Jelodar, Ali Rafati.

**Investigation:** Leili Valizadeh, Razieh Omidvar, Monireh Kamali.

**Resources: **Sepideh Taghavi, Marzieh Mirtajaddini, Nasim Naderi.

**Data Curation: **Monireh Kamali, Soroush Naseh, Leili Valizadeh, Razieh Omidvar.

**Writing—Original Draft Preparation: **Sepideh Taghavi, Hoda Raffiei Jelodar, Ali Rafati.

**Writing—Review and Editing: **Ali Rafati, Nasim Naderi, Ahmad Amin, Soroush Naseh.

**Supervision: **Sepideh Taghavi.

**Project Administration: **Hoda Raffiei Jelodar.

**Funding Acquisition: **not applicable.

## Funding

 None.

## Ethical Approval

 The RHC’s ethics review committee approved this study with the ethics code IR.RHC.REC.1400.076. We adhered to the declaration of Helsinki in this investigation. All patient records were kept confidential.

## Competing Interests

 The authors declare no conflict of interest.
